# Polymeric Nitrogen A7 Layers Stabilized in the Confinement of a Multilayer BN Matrix at Ambient Conditions

**DOI:** 10.1038/s41598-018-31973-7

**Published:** 2018-09-13

**Authors:** Xuhan Shi, Bo Liu, Shijie Liu, Shifeng Niu, Shuang Liu, Ran Liu, Bingbing Liu

**Affiliations:** 1 State Key Laboratory of Superhard Materials, Jilin University, Changchun, 130012 P.R. China; 2 School of Physics and Engineering, and Henan Key Laboratory of Photoelectric Energy Storage Materials and Applications, Henan University of Science and Technology, Luoyang, 471003 China

## Abstract

Polymeric nitrogen, as a potential high-energy-density material (HEDM), has attracted many theoretical calculations and predictions for its potential applications, such as energy storage, propellants and explosives. Searching for an effective method to stabilize polymeric nitrogen in ambient conditions of temperature and pressure has become a hot topic. Herein, we propose a new hybrid material where polymeric nitrogen layers are intercalated in a multilayer BN matrix forming a three-dimensional structure, by means of ab initio density functional theory. It is demonstrated polymeric nitrogen layers can be stable at ambient conditions and can release tremendous energy just above 500 K, more gentle and controllable. Further calculations reveal the new hybrid material exhibits a much smaller charge transfer than that of previous reports, which not only stabilizes polymeric nitrogen layer at ambient conditions, but also favours energy releasing at milder conditions. It is also very exciting that, the weight ratio of polymeric nitrogen in new material is up to 53.84%, approximately three times higher than previous one-dimensional hybrid materials. The energy density (5.4 KJ/g) also indicates it is a promising HEDMs candidate. Our findings provide a new insight into nitrogen-based HEDMs capture and storage.

## Introduction

As a potential high-energy-density material (HEDM), polymeric nitrogen has attracted increasing interest because of the widely applications in energy storage, rocket propellants and explosives, and it can be decomposed into pure inert gas N_2_ molecules which are environmentally friendly^1–3^. Polymeric nitrogen can be released a great amount of energy, once it dissociates into N_2_ molecules, which is at least three times higher than the most powerful energetic materials^4^. This is because nitrogen exhibits uniquely large difference in energy between that of the single bond (160 KJ/mol) or the double bond (418 KJ/mol) and the triple bond (954 KJ/mol)^1–4^. Over the years, many expectant forms of polymeric nitrogen have been proposed theoretically as targets for synthesis, such as chainlike structures^5^, layered structures^6–9^, and network structures^3^. Recently, several predictions have been proven in experiment^1,2^, among which the most prominent case is the so-called cubic gauche nitrogen (cg-N), connected with single bonds into a polymeric network, and successfully synthesized under extreme conditions (110 GPa, 2000 K)^1,10,11^. However, these obtained polymeric nitrogens cannot be quenched to ambient conditions. Therefore, searching for an effective method to stabilize polymeric nitrogen in ambient conditions of temperature and pressure has become a hot topic.

Recently, several theoretical calculations predicted that polymeric nitrogen chains become stable at ambient conditions while being confined in host materials, including carbon nanotubes^12^, silicon carbide nanotubes^13^, boron nitride nanotubes^14^ or multilayer graphene matrix^15^, and the stability can be explained by a charge transfer from the host materials to the guest N-chains. Experimentally, a N_8_-polynitrogen stabilized on the positively charged sidewalls of multi-walled carbon nanotubes (MWCNTs) has been synthesized using cyclic voltammetry (CV) under ambient conditions^16^. Indeed, all the previous attempts successfully stabilized polymeric nitrogen at ambient conditions by means of an effective nano-confinement strategy. But, we noticed from their data that the dissociation temperature of these hybrid systems is higher than 1400 K^14^, even above 5000 K^17^. Such a high temperature condition makes the energy releasing process very difficult and consuming large energy, which is not realistic for practical application. Therefore, seeking for a new hosting material to stabilize polymeric nitrogen at ambient conditions and to allow its energy releasing at a milder condition compared with previous reports is a great challenge. In addition, as a HEDM, the energy density of hybrid systems is strongly dependent on their weight ratio of polymeric nitrogen. However, the weight ratio of polymeric nitrogen in these predicted hybrid systems are usually less than 21%^12–15^. This much lower weight ratio of polymeric nitrogen will significantly affect overall energy of the hybrid materials thereby hinder their applications in reality. Thus, it is necessary to selecting a new hybrid material with high weight ratio of polymeric nitrogen. Moreover, previous studies have been limited on the stabilization of one-dimensional polymeric nitrogen chains. It is still unclear what behaviour of 2D layered polymeric nitrogen while actually be induced by the confinement in some template. In particular, it is still unknown whether or not the novel three-dimensional hybrid system will choose the known physical laws under confinement.

In this study, we theoretically propose a hybrid material, where polymeric nitrogen layers (A7)^7,18^ are sandwiched between multilayer BN sheets (A7@BNSs). Interestingly, finite temperature simulations demonstrate A7 layers become stable at ambient conditions, and dissociates into N_2_ molecules and release tremendous energy just above 500 K, more gentle and controllable compared with previous hybrid systems. Further calculations reveal A7@BNSs exhibits a smaller charge transfer than the previous reports. More importantly, the smaller charge transfer from BN sheet to A7 layer compared with previous works not only stabilizes A7 layer at ambient conditions, but also contributes to its energy releasing at milder conditions than their one-dimensional hybrid systems. Moreover, weight ratio of polymeric nitrogen in the new hybrid system (53.84%) is approximately three times higher than previous one-dimensional hybrid materials, leading to a satisfactory energy density (5.4 KJ/g), considerably higher than that of the modern explosive TNT (4.2 KJ/g).Thus, we present an effective approach for stabilizing nitrogen-based HEDMs and controlling their energy releasing.

## Computational Method

The theoretical calculations in this work are performed within density functional theory (DFT) approach, and generalized gradient approximation (GGA) PBE (Perdew-Burke-Ernzerhof)^19^ is used to describe the exchange-correlation interactions. The exchange and correlation effects are described here by GGA instead of LDA because we use pure h-BN structure as our template to check two functions which is more accurate. We optimized the pure h-BN template structure with LDA and GGA exchange functions and compared the parameters with experimental values for pure h-BN and we found that GGA function can fit experimental results well. Our geometry relaxation and ab initio Molecular Dynamic simulations are applied with the projector augmented plane-wave (PAW) method^20^ as implemented in the Vienna ab initio Simulation Package (VASP) code^21^. When relaxing this system, we adopt a plane-wave basis with 600 eV energy cutoff and 16 × 16 × 5 Monkhorst Pack grid^22^ spacing to guarantee that the force on each atom is less than 0.05 eV/Å, while the energy convergence criteria of 1 × 10^−5^ eV are met. In order to further research the interaction between host material (BN matrix) and A7 layers, van der Waals interactions (optB88-vdW) are considered. The whole system is fully relaxed both with respect to cell shape and internal atomic positions with the fine convergence. We apply CASTEP code^23^ in Material Studio to verify the electronic properties of this hybrid system. Both of A7 and BN are crystallized in hexagonal phase, with the graphite-like structures, similar lattice parameters. The unit cell of this hybrid material contains one elementary unit cell of A7 layer (2atoms) and one BN sheet (2atoms). A commensurability condition is adopted in the plane of nitrogen layer, taking four unit cells of nitrogen layer and four unit cells of BN sheet in the same directions. The lattice mismatch δ = |(4* L_A7_-4*L_BN_)/L_BN_| = 0.034; L refers to lattice constants a and b. |δ| <5%, the lattice matches well, which allows keeping the system size at computationally affordable level. Molecular Dynamics simulations are performed in the canonical (constant volume and constant temperature) ensemble with a supercell of 128 atoms for whole 2 ps time period, 1 fs time step. During simulations, the temperature is set to 300–2000 K with rough 200 K or fine 50 K spacing step by using Nose thermostat. The supercell with periodic boundary conditions of 128 atoms is used to calculate band structures, density of states and electron density distribution with a denser k-point grid, a spacing of 2π × 0.015 Å^−1^ was used to sample the Brillouin zone. The Bader charge analysis^24,25^ reveals the amount of charge transfer between BN sheet and A7 layer. The phonon frequencies are calculated using the supercell method with the PHONOPY code^26^.

## Results and Discussion

**Figure 1:**
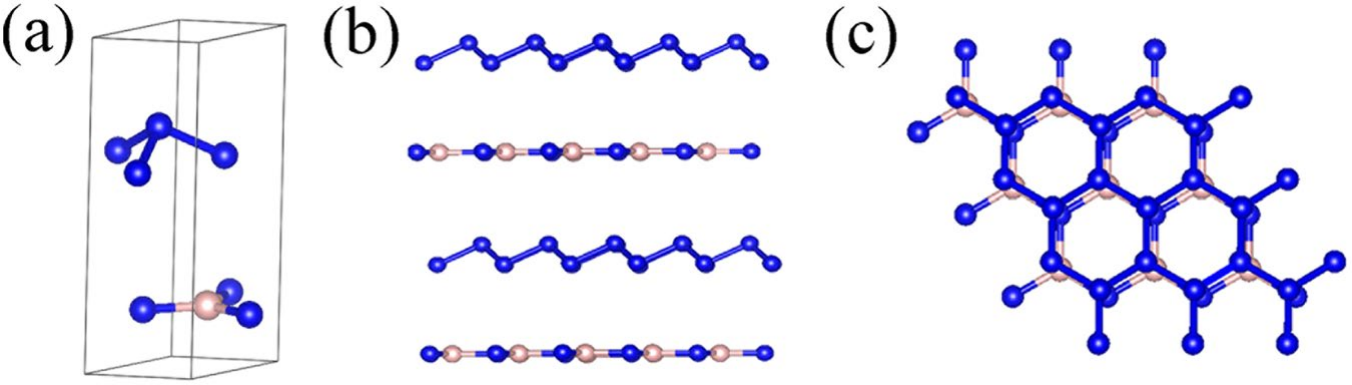
The unit cell of A7@BNSs system (**a**), the view of perpendicular to stacking direction (**b**) and the stacking direction of A7@BNSs (**c**). Blue is for nitrogen atoms, and pink is for boron atoms in the figure.

We insert polymeric nitrogen A7 layers into a multilayer BN matrix, forming a new three-dimensional hybrid material (A7@BNSs). To find the lowest energy configuration of A7@BNSs, different overlapped initial structures are used as starting configuration to fully relax at ambient pressure. Fig. 1 shows the final lowest total-energy configuration of A7@BNSs obtained by mean of geometry optimization. Fig. 1(a) is the obtained lowest energy unit cell of A7@BNSs, and the different views of A7@BNSs supercell are shown in figure (b,c). Our geometry relaxation calculations show the N-N bond length of polymeric nitrogen in A7@BNSs is 1.578 Å, which is similar to the bond length of initial A7.^7^ The optimized system exhibits that BN sheet overlap A7 layer with about 4 Å interlayer distances (3.6 Å and 4.3 Å, respectively). The unit cell lattice constants of this hybrid system are listed as follow: a = b = 2.46 Å, c = 7.41 Å; α = 87.4°, β = 92.6°, γ = 120°. The volume of unit cell is 38.81 Å^3^. The simulation cell adopts a 4 × 4 × 2 supercell, containing 128 atoms. a = b = 9.74 Å, c = 15.97 Å; α = 88.2°, β = 92.2°, γ = 120°.

**Figure 2:**
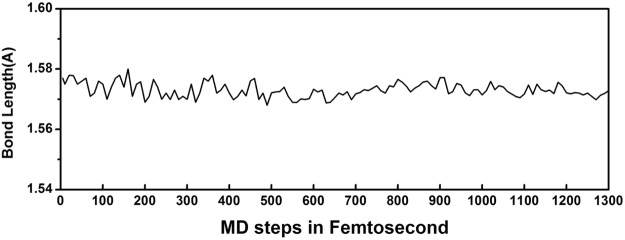
The evolution of single N-N bond length as time at ambient conditions in NVT MD simulation.

Thermal stability of hybrid system is examined via constant-temperature constant-volume Molecular Dynamics (MD) simulations, adopting 4 × 4 × 2 supercell containing 128 atoms. Fig. 2 exhibits the evolution of single N-N bond length as time at ambient conditions in NVT MD simulation. From Fig. 2, we find there is no bond-rupture in the whole MD simulation process, but only some slight fluctuations. The overall hybrid system is well kept after 2 ps simulation. This indicates the polymeric nitrogen layer confined in a multilayer BN matrix, can keep its configuration stable at ambient conditions. We also perform a MD simulation of isolated polymeric nitrogen at 300 K for comparison with a supercell for whole 7 ps time period in NVT ensemble and the result shows that A7 layers break down into molecular nitrogen, which means isolated A7 cannot be stable under ambient conditions.

**Figure 3:**
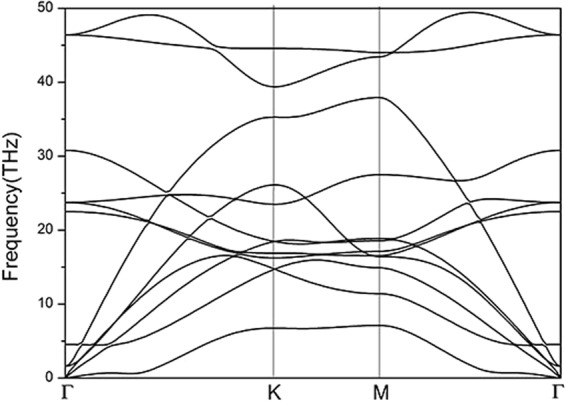
Phonon dispersion curves of the A7@BNSs at ambient pressure.

In order to confirm the stability of A7@BNSs, we calculated phonon spectrum for A7@BNSs by the supercell method with the PHONOPY code at ambient pressure. The phono dispersion relationship of A7@BNSs is shown in Fig. 3. There is no imaginary frequency shown in the whole Brillouin zone, which intuitively indicates the A7@BNSs system is dynamic stable at ambient pressure.

**Figure 4:**
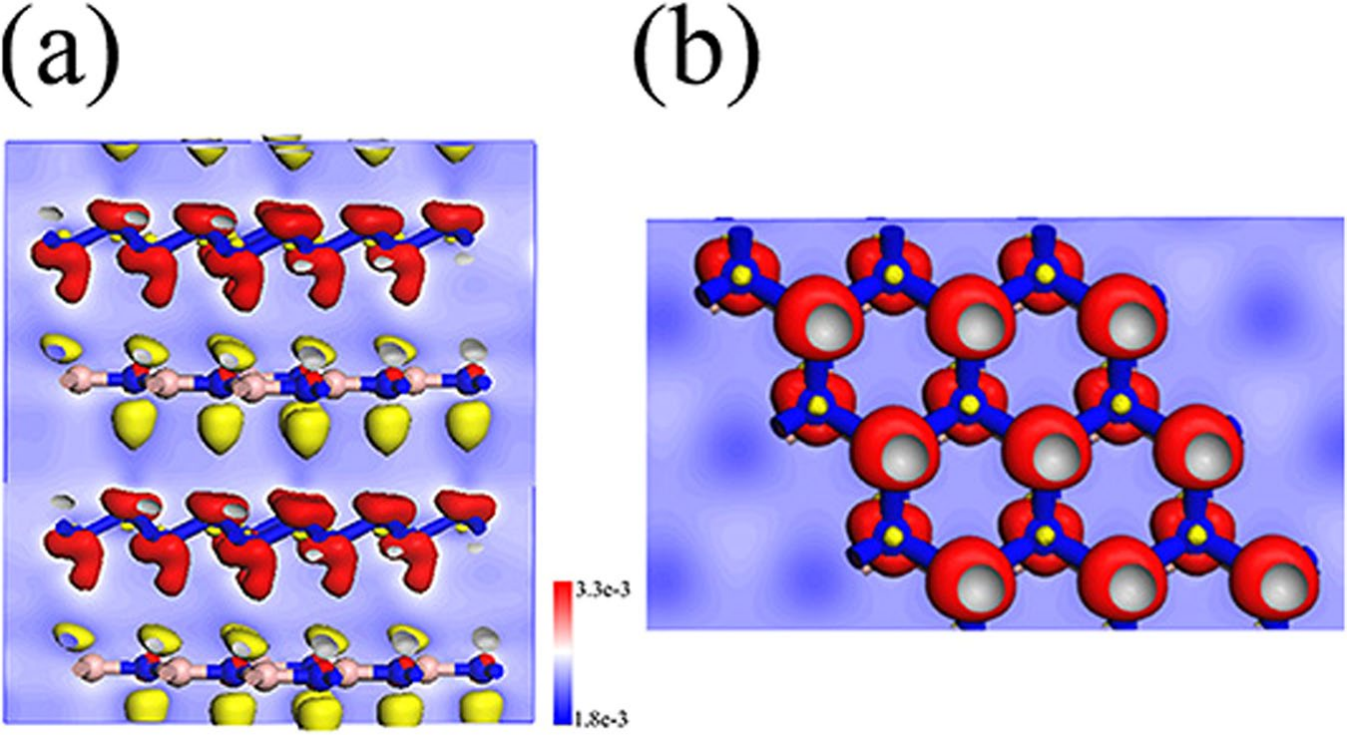
The electron density difference for A7@BNSs at ambient pressure. Electronic density of the A7@BNSs system, minus the sum of electronic densities of isolated nitrogen layer and boron nitride sheet. The red and yellow colors indicate the electrons accumulation and depletion, respectively. The unit is e/Å^3^. Blue denotes nitrogen atoms, and pink denotes boron atoms.

To understand the physical mechanism leading to the stability of the A7@BNSs system, we carried out the electronic properties analysis. The electron redistribution of the A7@BNSs system is calculated by subtracting the electron of A7 layer and BN sheet from the electronic density of A7@BNSs.The electron density difference is plotted in Fig. 4. Obviously, the electron density is increased around A7 layer and decreased around BN sheet. Thus, BN sheet transfers a small amount of electrons to A7 layer. Bader charge transfer analysis accurately reveals that 0.26 electrons are transferred in this A7@BNSs supercell system, about 0.004 electrons transfer to each polymeric nitrogen atom, strongly consistent with the charge redistribution calculation. The small charge transfer leads to the accumulation of negative charges on A7 layer and positive charges on BN sheet, respectively, which gives rise to a Coulombic interactions and then significantly favor the stabilization of polymeric nitrogen layers at ambient conditions. A similar stabilization mechanism also takes place in the case of polymeric nitrogen chains confined in CNTs^12^, SiCNTs^13^, BNNTs^14^, and a multilayer graphene matrix^15^.

**Figure 5:**
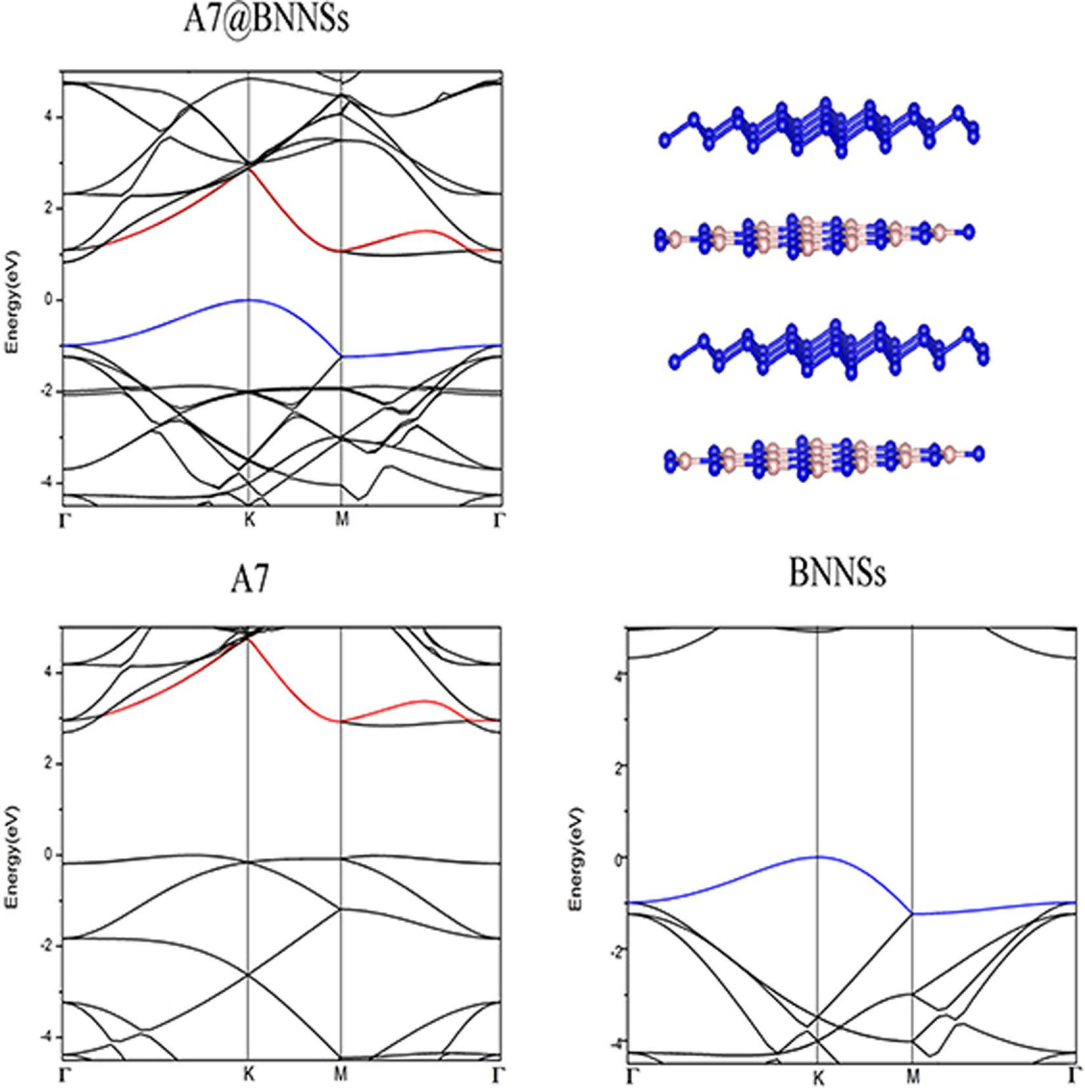
Band structures for the A7@BNSs system (**a**), isolated A7 layer (**b**), and isolated BN sheet (**c**) at ambient pressure, respectively.

We further studied the interaction between the BN sheet and the A7 layer by calculating band structure of this hybrid system. To highlight the effects of the interaction between A7 layer and BN sheet, the band structures for an isolated A7 layer and an isolated BN sheet also calculated for sake of comparison, using the same atomic coordinates as in the A7@BNSs system. Fig. 5 shows the band structures for the A7@BNSs system, the isolated A7 layer and the isolated BN sheet, respectively. By comparing these band structures in Fig. 5, we find in the energy range from −4 to + 4 eV, the band structure of A7@BNSs hybrid system is formed by the superposition energy bands of the A7 layer and the BN sheet: the top of valence band of A7@BNSs comes from the BN sheet, and the bottom of conduction bands comes from the A7 layer. At the same time, it is noticed the conduction bands of A7 layer in hybrid system shift downward about 1.8 eV toward to fermi level compared with isolated A7 conduction bands and the conduction bands of BN sheet shift upwards compared to isolated BN sheet. This phenomenon reveals that there is a weak interaction between the nitrogen layers and BN sheets in A7@BNSs system.

**Figure 6:**
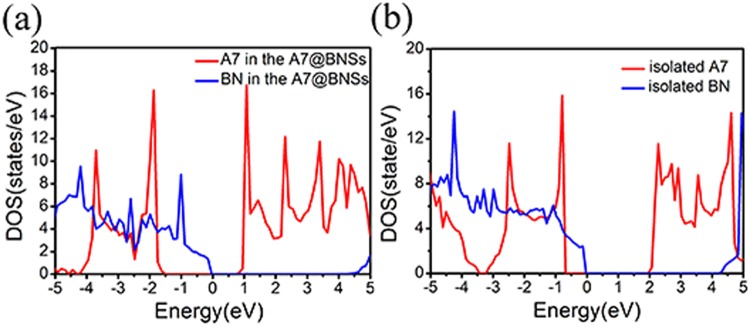
Projected density of electronic states for the A7 layer and BN sheet in A7@BNSs system (**a**) and projected density of electronic states for the isolated A7 layer and isolated BN sheet (**b**).

**Figure 7:**
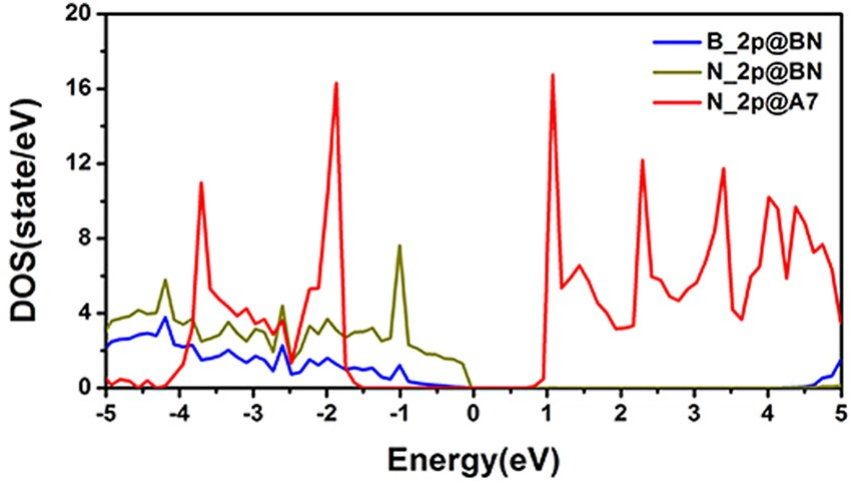
Density of states for A7@BNSs system projected on atomic orbitals.

The hybridization between A7 layer and the BN sheet can be clearly seen through the electronic density of state (DOS) calculations. Fig. 6 shows the DOS for A7@BNSs system, projected on the orbitals of A7 layer atoms, as well as on the orbitals of the BN sheet. Density of states for the isolated A7 layer and BN sheet are shown for comparison. From Fig. 6, we observe the corresponding DOS peaks of BN sheet in A7@BNSs downshift compared with the isolated BN sheet, while the peaks of A7 layer in A7@BNSs upshift compared with the isolated A7 layer. Further comparing these DOSs in Fig. 6(a and b), a weak hybridization between A7 and BN in A7@BNSs system can be clearly seen at about −1.87 eV, −2.2 eV, −2.47 eV below fermi energy (E_F_), while there is no such phenomenon can be seen in isolated BN and A7 layers. These changes of DOS of the A7 layer and BN sheet in A7@BNSs originate from the weak hybridization between A7 and BN, leading to the charge transfer and stabilization of A7@BNSs system. More details about hybridization can be presented by partial density of states (PDOS) calculations projected on atomic orbitals for A7@BNSs system. As shown in Fig. 7, the PDOS results not only verify that the hybridization is located at around 3 eV below E_F_, which is significantly consistent with Fig. 6. More importantly, the PDOS results reveal that the weak hybridization attributes to the B_2p, N_2p orbitals in BN sheet and N_2p orbital in A7 layer.

**Figure 8:**
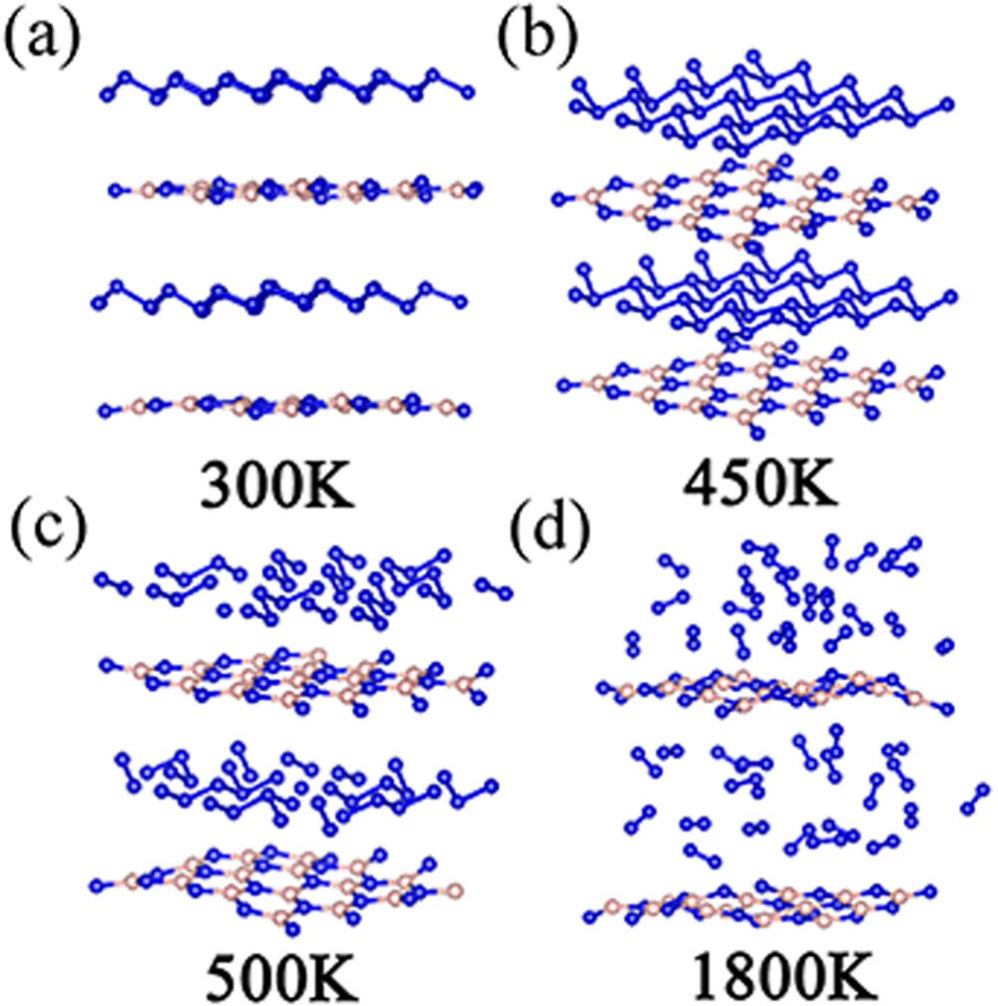
Transformation path of A7@BNSs to N_2_@BNSs at high pressure, (**a**) the initial state A7@BNSs at ambient temperature; (**b**) the intermediate stable state of A7@BNSs; (**c**,**d**) the dissociation state and the final state (N_2_@BNSs) at 500 K and 1800K, respectively.

In addition, we explored the stable temperature range of A7@BNSs system performed by MD simulations from 300 K to 2000K for 2 ps time period. According to the simulations results, the N-N bond in A7 remain continuous up to 450 K, indicates that A7 layer can keep its structure stable in the range of 300 K~450 K, while dissociate into N_2_ completely at 1800 K. Moreover, snapshots of the transformation pathway are shown in Fig. 8. We find polymeric nitrogen layer (A7) transforms into different lengths of N-chains and nitrogen molecules just at 500 K. With increasing temperature up to 1800 K, nitrogen chains dissociate into nitrogen molecules completely. Interestingly, as shown in Table 1, the dissociation temperature of polymeric nitrogen in the new hybrid system is much milder than polymeric nitrogen chains which confined both in BNNTs (1400 K)^14^ and CNTs (5000 K)^12^. To understand the observed difference, we compared the amount of charge transfer of A7@BNSs and other hybrid systems^12,14^. From Table 1, we can see the amount of charge transfer in A7@BNSs is only 0.26e (0.004e/N atom), much smaller than that of N_8_@BNNT^14^ and N_8_@CNT^12^. Interlayer interaction is the stabilizing factor and it is related to charge transfer. The more charge transfer, the stronger interaction between layers, the more stable hybrid system and the high stability leads to the higher decomposition temperature of polymeric nitrogen. This lower charge transfer compared with theirs leads to weaker interlayer interaction and lower stability than N8@CNTand N8@BNNT systems when heating hybrid systems, which results in lower decomposition temperature systems. In other words, the smaller charge transfer in hybrid system, the weaker interaction between host material and polymeric nitrogen, the lower decomposition temperature, thereby allows its energy releasing milder and controllable compared with previous reports.

**Table 1:** The amount of charge transfer and the dissociation temperature in A7@BNSs, N_8_@BNNTs and N_8_@CNTs hybrid systems.

Hybrid System	Charge Transfer	Dissociation Temperature
N_8_@CNTs^12^	0.4e (0.05 e/N atom)	>5000 K
N_8_@BNNTs^14^	0.32e (0.04 e/N atom)	1400 K
A7@BNSs	0.26e (0.004 e/N atom)	500 K

Finally, we found the weight ratio of polymeric nitrogen in A7@BNSs is up to 53.84%, approximately three times higher than previous one-dimensional hybrid materials^12–15^. Subsequently, we researched energy density for this new hybrid material at the PBE-GGA level. The initial and final states are accurately optimized to their equilibrium structures. The results show the energy gap between A7@BNSs and its decomposition products (N_2_@BNSs) is 2.9ev per unit cell, corresponds to an energy density of 5.4KJ/g, significantly higher than that of the modern explosive TNT (4.2KJ/g). This system proposed by us is a theoretical model, which can provide a possibility for the polymeric nitrogen confinement especially for layer materials. Experimentally, a possible way to overcome the energy barrier may be the application of external pressure and temperature on the hybrid N_2_-BNSs system. Therefore, the new A7@BNSs hybrid structure can be a possibility for future HEDM candidate.

## Conclusion

In summary, we have proposed a new hybrid material, where polymeric nitrogen layers are intercalated in a multilayer BN matrix by *ab initio* DFT approach. The results have demonstrated polymeric nitrogen layers can be stable at ambient conditions, while confined in a multilayer BN matrix. The stabilization mechanism originates from the small charge transfer from BN sheet to polymeric nitrogen layer. Finite temperature molecular dynamics simulations show polymeric nitrogen layers in A7@BNSs dissociate into N_2_ and release energy just at above 500 K. More importantly, the A7@BNSs has a small charge transfer between A7 layer and BN sheet and this small charge transfer stabilizes A7 layer at ambient conditions. Comparing with previous hybrid systems, our system has a smaller charge transfer than theirs, which leads to weaker interlayer interaction and a lower stability which results in lower decomposition temperature, contributes to its energy releasing at a milder condition. Moreover, the weight ratio of polymeric nitrogen in the new hybrid system is 53.84%, approximately three times higher than previous one-dimensional hybrid materials, leading to a satisfactory energy density (5.4KJ/g), considerably higher than that of the modern explosive TNT (4.2KJ/g). Our findings open a new avenue to capture nitrogen-based HEDMs.

## Data Availability

All data generated or analysed during this study are included in this published article (and its supplementary information files).
